# Growth of Deciduous and Evergreen Species in Two Contrasting Temperate Forest Stands in Korea: An Intersite Experiment

**DOI:** 10.3390/plants11070841

**Published:** 2022-03-22

**Authors:** Byung Bae Park, Youngtak Ko, Jonathan O. Hernandez, Ser-Oddamba Byambadorj, Si Ho Han

**Affiliations:** 1 Department of Forest and Environmental Sciences, Chungnam National University, Daejeon 34134, Korea; forest.kyt@gmail.com (Y.K.); johernandez2@up.edu.ph (J.O.H.); seroddamba@gmail.com (S.-O.B.); bupleurumhan@cnu.ac.kr (S.H.H.); 2 Department of Forest Biological Sciences, University of the Philippines, Los Baños 4031, Philippines

**Keywords:** coniferous forest, broadleaved forest, *Fraxinus rhynchophylla*, light availability, seedling community, shade-tolerant, soil texture, understory, *Zelkova serrata*

## Abstract

Poor seedling establishment and growth can be a result of the limitation of light and soil resources in the forest understory. Here, we investigate the interacting effects of stand and soil characteristics on the seedling growth of deciduous species (*Fraxinus rhynchophylla* and *Zelkova serrata*) and evergreen species (*Pinus koraiensis*) through a 3-year intersite experiment in two contrasting forest stands. Seedlings were grown in both oak and pine stands using two different soil types, i.e., gray-brown forest soil (GB) and red-yellow forest soil (RY). Soil physicochemical properties, light intensity, tree-seedling height, root-collar diameter (RCD), and biomass growth were analyzed between two stands and/or soil types. Light availability was generally more abundant in the pine stand (mean: 1074.08 lx or 20.25%) than the oak stand (mean: 424.33 lx or 9.20%) throughout the year. The height and RCD growth of fast-growing and deciduous *F. rhynchophylla* and *Z. serrata* were higher in the pine than in the oak stand, particularly in GB soil. The growth of the slow-growing and evergreen *P. koraiensis* was not affected by the forest stand, except for its higher root growth in the oak stand and RY soil. Therefore, abundant light availability can enhance the growth and seedling establishment of *F. rhynchophylla* and *Z. serrata* in the pine-stand understory. Contrarily, *P. koraiensis* may be planted in the understory regardless of light condition, but with a slower growth rate.

## 1. Introduction

Tree-seedling establishment and juvenile growth of newly germinated seeds are critical life-history stages of trees, because survival and growth performance at the seedling stage affect the succession, structure, and function of forest ecosystems [1,2]. Poor seedling-community establishment and growth can be a result of resource limitation and heterogeneity of light and soil resources in the forest [3]. Seedlings of different species and life-history traits are thus expected to respond differently to resource limitation and heterogeneity due to variation in growth potential and shade tolerance [4]. In temperate forests, trade-offs between survival and growth along resource gradients are well-documented for saplings, but there is less evidence available for the seedling stage [5,6,7]. Thus, studies on seedling growth of contrasting species with different requirements for essential resources can help us understand how seedlings perform relative to each other via inter-site conditions.

The amount of light is one of the most important and heterogeneous factors influencing plant recruitment, survival, and growth, particularly of the newly established seedlings in the forest understory. The extent of the effects of light on tree seedlings’ overall performance in the understory may be influenced by the type of forest. In a deciduous forest, trees lose their leaves in the fall, the canopy opens, and abundant light begins to penetrate the forest floor; and then during spring and summer, they form a dense layer of canopy leaves which again blocks most of the sunlight. Such a characteristic of a deciduous forest plays a crucial role in the regeneration and early growth of woody species in the understory through the canopy-gap heterogeneity. For example, the seedling density and growth of hardwood species increases with increases in the canopy gap, which influences the availability of microsites [8]. Contrarily, previous studies have reported that coniferous trees significantly decrease light availability to the understory, resulting in a considerable reduction in vascular plant growth [9]. Broadleaf canopy favors the growth and establishment of full-sun plants, whereas evergreen coniferous trees increase the abundance of shade-tolerant plants [10,11]. Plant responses to contrasting light availability within a single site are well-documented in tropical rainforests [12], but to the best of our knowledge, only a few intersite experiments have been conducted in temperate regions to date. A better understanding of the seedling growth of fast-growing and slow-growing species in different forest types can help inform forest-management practices in temperate zones.

The characteristics of soil are also an important factor influencing seedling growth and establishment [13]. Soil texture affects the root growth and biomass allocation of fast-growing *Prosopis* seedlings, such that root growth is greater in sandy soils than loamy soils and aboveground growth is higher in clayey soils than other texture classes [14]. Similarly, well-drained sandy soils were found to be ideal for the seedling growth and establishment of legume species due to good aeration and drainage [15]. In Scots-pine stand, soil mineral contents also showed a positive correlation with seedling-height growth [16]. Further, the interaction of soil characteristics and light availability was a significant environmental factor that influenced vegetation growth in a mixed-conifer understory [17]. While light is deemed the most important resource for seedling growth [18], different soil characteristics and tree species have not yet been fully investigated simultaneously with light under intersite conditions.

It has long been recognized that there exist life-history trade-offs between full-sun and shade-tolerant tree species in their degree of dependence on light for growth, survival, and establishment in the understory [19]. For example, the reduction in seedling height and biomass due to shade was greater in full-sun, fast-growing pioneer species compared with slow-growing shade-tolerant ones [20]. Fast-growing species are more plastic to changing environments, enabling them to expedite growth rates in high-light conditions. Thus, in this study, we used three species that have contrasting life-history traits: namely, *Fraxinus rhynchophylla* Hance, *Pinus koraiensis* Siebold & Zucc., and *Zelkova serrata* (Thunb.) Makino. Two of these species are broadleaved deciduous (i.e., *F. rhynchophylla* and *Z. serrata*), while the other one is a needle-leaved evergreen tree species (i.e., *P. koraiensis*). These species are co-occurring in the forest; thus, the differences in their responses to resource availability in the forest understory may have important implications for forest management.

In this study, we investigated the seedling growth of fast-growing and deciduous *F. rhynchophylla* and *Z. serrata* and slow-growing and evergreen *P. koraiensis* in different forest stands with contrasting light conditions and soil characteristics through an inter-site experiment. We hypothesized that (1) seedling growth of fast-growing and deciduous species are higher in needle-leaf stands or in sites with higher light availability than oak stands or sites with lower light availability; (2) seedling growth of slow-growing evergreen species are unaffected, regardless of light and soil conditions; and (3) the effects of soil attributes on seedling growth vary significantly by forest stand.

## 2. Results

### 2.1. Variation in Soil Characteristics and Light Availability in Oak and Pine Forest Stand

Soils from the oak stand and pine stand were matched to gray-brown forest soils (GB) and red-yellow forest soils (RY), which were filed by the National Institute of Forest Science in Korea. The present study showed that the interaction effects of forest stand and soil horizon on the soil’s physicochemical characteristics were not statistically significant (Table S1). However, the main effects of either stand and soil horizon on OM, TN, exchangeable K^+^ and Mg^2+^, and CEC were statistically significant between GB and RY soil and between A- and B-soil horizons (Table 1 and Table S1). OM, TN, and CEC were significantly higher in RY soil than that GB soil. Contrarily, the GB soil had a significantly higher exchangeable K^+^ and Mg^2+^ than the RY soil. Soil pH and sand proportion were significantly higher in the GB than the RY soil. On average, the A-soil horizon had a considerably higher OM, TN, exchangeable K^+^ and Mg^2+^, and CEC than the B-soil horizon in both GB and RY soils.

**Figure 1:**
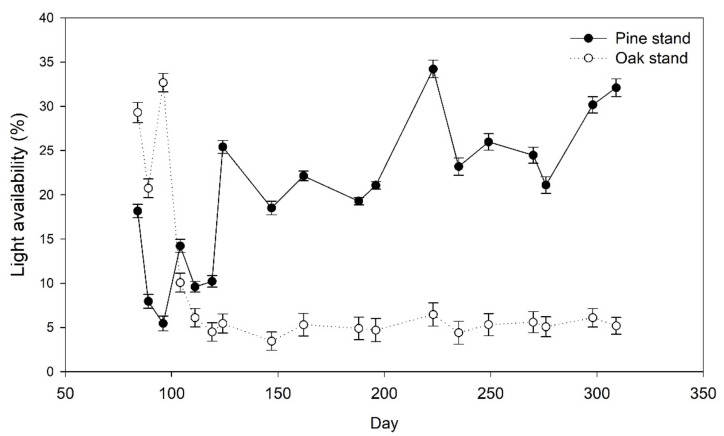
Light availability (%) at oak and pine stands measured during the onset of leaf flush until mid-November.

Relative light availability was higher in the pine stand (mean: 1074.08 lx or 20.25%) than the oak stand (mean: 424.33 lx or 9.20%) throughout the year (Figure 1). During growing season, light availability was higher in the pine stand than the oak stand because light interception was lower in the former stand than in the latter stand as light intensity increased from April and May to August (Figure 1).

### 2.2. Height Growth, RCD Growth, and Biomass of Fraxinus rhynchophylla, Pinus koraiensis, and Zelkova serrata between Forest Stands and Soil Types

**Figure 2:**
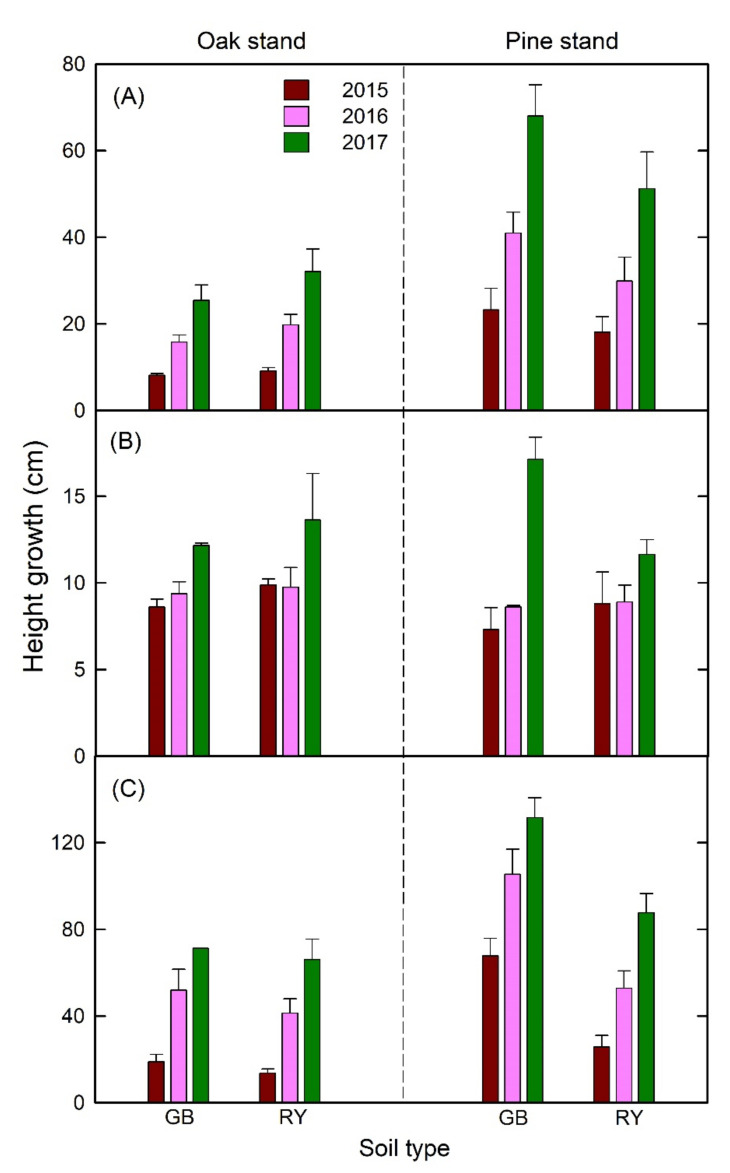
Height growth of (**A**) *Fraxinus rhynchophylla*, (**B**) *Pinus koraiensis*, and (**C**) *Zelkova serrata* in different soil and forest stands from 2015 to 2017.

There were no significant interaction effects of stand × soil treatments on the height growth of all species, but the effect of time × stand in *F. rhynchophylla* was significant (Table S2). The main effect of forest stand on the height growth was also significant in both fast-growing and deciduous *F. rhynchophylla* and *Z. serrata*, but not in the slow-growing and evergreen *P. koraiensis* (Figure 2 and Figure S2; Table S2). Specifically, the relative height growth of the seedlings was generally higher in the pine stand compared with oak stand for *F. rhynchophylla* and *Z. serrata.* In the pine stand, the height of *F. rhynchophylla* and *P. koraiensis* seedlings generally increased with time by 13–73% and 15–56%, respectively (Figure 2 and Figure S2). In contrast, the relative height growth of *Z. serrata* declined with time by 22–80% in the oak stand, but when it was planted in the pine stand using RY soil, the relative height growth significantly increased by 58% in the 2015–2016 period (Figure 2 and Figure S2). In this study, a significant effect (*p* = 0.029) of soil on the height growth of *Z. serrata* was also detected: GB generally had a higher height growth than the RY soil type in both oak and pine stands (Figure 2; Table S2).

**Figure 3:**
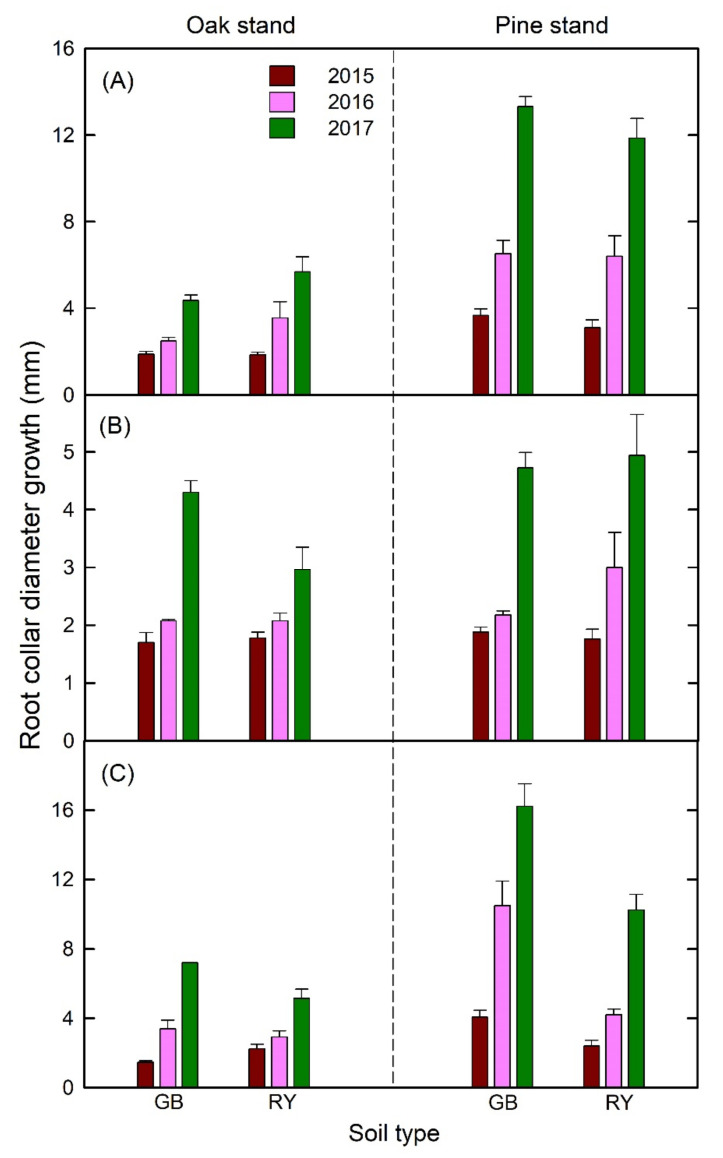
Root-collar-diameter growth of (**A**) *Fraxinus rhynchophylla*, (**B**) *Pinus koraiensis*, and (**C**) *Zelkova serrata* in different soil and forest stands from 2016 to 2017.

The effect of time × stand interaction on RCD was significant for *F. rhynchophylla* and *Z. serrata* (Figure 3; Table S2). Similar to relative height growth, the pine stand resulted in a significantly higher relative RCD growth rate than the oak stand for the two fast-growing deciduous species, especially in the 2016–2017 experimental period (Figure 2 and Figure S3). The marginal interaction effects of time × soil and stand × soil on the RCD of *Z. serrata* were also detected (Table S2). Specifically, GB resulted in a higher RCD compared with RY in the pine stand and the 2016–2017 period (Figure 3). Similar to height, the RCD growth of *P. koraiensis* was significantly influenced by time, but no stand or soil-treatment effects were observed. The relative RCD of *P. koraiensis* increased with time regardless of forest stands and soil types.

**Figure 4:**
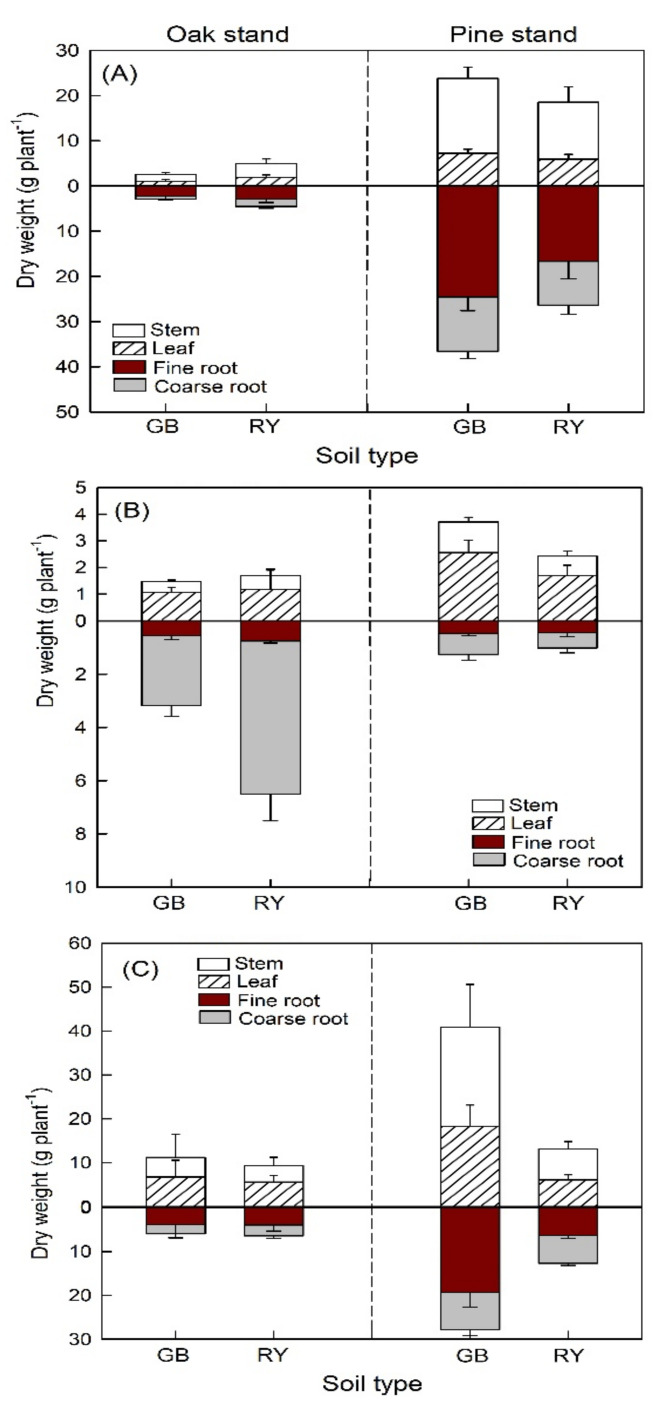
Biomass growth of (**A**) *Fraxinus rhynchophylla*, (**B**) *Pinus koraiensis*, and (**C**) *Zelkova serrata* in different soil and forest stands at harvesting year.

No significant interaction effect of stand × soil type was detected on biomass growth of *F. rhynchophylla* and *P. koraiensis*, but a significant interaction effect was found in the case of *Z. serrata* (Table S3). Specifically, *Z. serrata* seedlings grown in the pine stand using the GB soil type showed better biomass growth than the oak stand (Figure 4). Here, the pine stand yielded a significantly higher biomass growth of *F. rhynchophylla* than the oak stand regardless of soil type (Figure 4). There were contrasting patterns among species in biomass allocation between above-ground and below-ground: the oak stand resulted in a significantly higher root–shoot ratio of *P. koraiensis* than the pine stand in all soil types, but the opposite direction was observed for *F. rhynchophylla* and *Z. serrata*.

### 2.3. Principal Component Biplot Analysis

**Figure 5:**
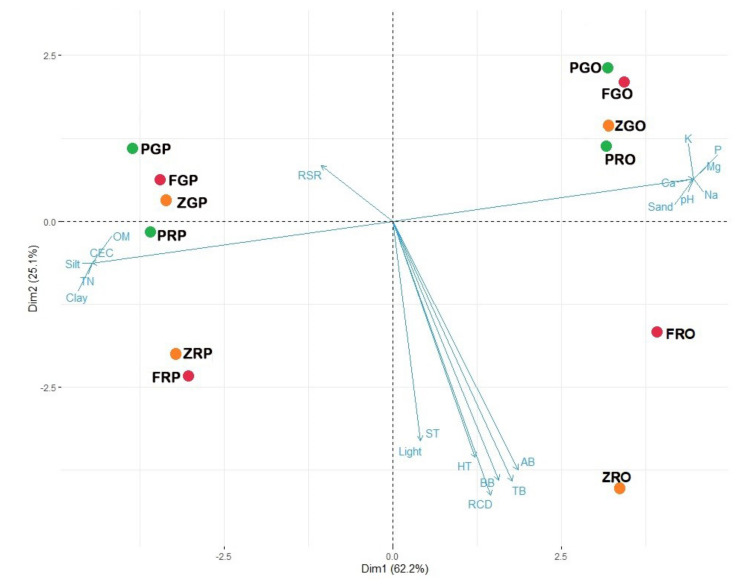
Principal-component-loading plot of the growth and site variables and individuals of *Fraxinus rhynchophylla* (FGP, FGO, FRO, FRP), *Pinus koraiensis* (PRP, PGP, PRO, PGO) and *Zelkova serrata* (ZGP, ZRO, ZRP, ZGO) planted in oak and pine stands. RCD—root collar diameter, HT—height, BB—below-ground biomass, AB—above-ground biomass, TB—total biomass, RSR—root-to-shoot ratio, ST—soil temperature, TN—total nitrogen, CEC—cation-exchange capacity, OM—organic matter, pH—soil pH, Na—exchangeable sodium, Mg—exchangeable magnesium, P—available phosphorus, Ca—exchangeable calcium, and K—exchangeable potassium.

We performed PCA to determine the relationship of the growth, light availability, and soil variables measured in *F. rhynchophylla*, *P. koraiensis*, and *Z. serrata* across forest-stand and soil-type treatments (Figure 5). The first two components accounted for 87.3% of the variation in the dataset. Specifically, Dim1 accounted for 62.2% of the variation and was highly related to most of the soil variables, represented mostly by seedlings planted in pine soil on the left side and by seedlings grown using oak soil on the right side of the biplot. *P. koraiensis* was strongly influenced by soil characteristics rather than stand characteristics. *F. rhynchophylla* and *Z. serrata* are influenced by both the soil and stand characteristics. Dim2 accounted for 25.1% of the variation and was highly related to growth variables, light availability, and soil temperature (ST). Light and ST were highly correlated with height, RCD, and biomass growth.

## 3. Discussion

Based on our first hypothesis, we found that the fast-growing, deciduous *F. rhynchophylla* yielded higher relative height and RCD growth in the pine stand than the oak stand, with light intensities ranging from 1001.16–1147.01 lx and 396.67–452.66 lx, respectively. Although the effects of soil on height and RCD growth rates were not significant based on the results of the ANOVA, the PCA revealed that *F. rhynchophylla* seedlings (i.e., ZRP) planted in RY soil were highly associated with OM, TN, and CEC, whose concentrations were significantly higher in the pine stand than the oak stand. This suggests that the higher annual light availability and/or OM, TN, and CEC in the pine stand may have increased the growth of *F. rhynchophylla.* This can also be supported by the observed high correlation between soil temperature (ST) and light availability. The higher soil temperature in the pine stand due to higher light penetration may have facilitated decomposition of organic matter, resulting in higher CEC. A study showed that soil temperature can regulate root–soil interactions by influencing the rate of litter decomposition and soil microorganisms’ activity [21]. Contrarily, the PCA biplot showed that the *F. rhynchophylla* seedlings planted in the oak stand (i.e., FRO) were highly associated with K^+^, Ca^2+^, Mg^2+^, and soil pH instead of light availability, suggesting that the lower light availability in the oak stand may have resulted in a lower RCD growth of *F. rhynchophylla* seedlings. Our findings support the results of Yan et al. [22], who reported the ability of *F. rhynchophylla* to regenerate naturally in a completely cleared and degraded forest. Any silvicultural systems that can provide species with abundant sunlight (e.g., clear-cutting) have also been recommended for *Fraxinus* species [23]. For example, the understory of *Larix kaempferi* plantations was dominated by *F. rhynchophylla* seedlings after thinning [24]. Further, a previous study reported the increasing trend in the density of shade-intolerant *F. rhynchophylla* from the forest understory to large gaps [25]. This confirms the observed lower height, RCD, and biomass growth of the species in the broadleaf oak stand, with lower light availability throughout the year. Lastly, it has long been reported that seedlings of *Fraxinus* species develop best in at least 45% full sunlight [23].

Neither soil nor stand characteristics significantly impacted the height and RCD growth of *P. koraiensis*, and this contradicted our second and third hypotheses. This suggests that other factors may have also influenced the growth of the species. Here, we found that the relative RCD of *P. koraiensis* increased with time in all forest stands and soil types. Results can be attributed to the functional type (i.e., slow-growing and evergreen) and shade tolerance of *P. koraiensis*. Many slow-growing evergreen conifers have low photosynthetic capacity and low nitrogen concentrations but with a long leaf lifespan, which is typical of tree species growing in environments unsuited for rapid growth [26]. A previous study revealed that slow-growing species in the temperate forest grew slowly or remained unaffected regardless of light condition (high, moderate, low levels) [6]. Similarly, Valladares et al. [27] reported that the two evergreen tree species exhibited a reduced growth due to their low light response to light and nutrient availability. The authors further attributed the results to the conservative resource-use strategy typical of evergreens. Young *P. koraiensis* seedlings may have exhibited such a conservative strategy, particularly during the initial months of the experiment because of the need to allocate more biomass to persistent tissues necessary for shade tolerance. In this study, we found a significantly higher root–shoot ratio (RSR) and coarse root of *P. koraiensis* in the oak stand (with lower light availability) than pine stand in all soil types. The PCA biplot also showed that soil characteristics predominantly influenced *P. koraiensis*’s RSR, which is negatively correlated with aboveground growth. Several studies have long reported that the higher root–shoot ratios of shade-tolerant species compared with shade-intolerant ones are an important trait related to shade tolerance [1,28]. A similar study also found a significantly higher biomass allocation to coarse roots of shade-tolerant *Fagus* and *Acer* species compared with other shade-intolerant species when planted under shade conditions [29]. This may have allowed the establishment of *P. koraiensis* seedlings that are less susceptible to resource limitations, i.e., soil nutrients and light in the oak stand, by capturing below-ground resources more effectively. Furthermore, plants adapted to low-resource environments, such as evergreen trees, are expected to exhibit a conservative resource-use strategy at the expense of fast growth [30]. This could further explain the observed insignificant effect of the stand and soil and their interactions on the height and RCD growth of *P. koraiensis*.

The results we observed in the fast-growing and deciduous *Z. serrata* also supported our first hypothesis, such that its seedlings grew well in the pine stand, with higher light availability throughout the year. Similar to *F. rhynchophylla, Z. serrata* seedlings did not grow well in the oak stand. The effect of light was also seen in the significant decrease in relative height growth in the 2016–2017 experimental periods in both forest stands, possibly indicating an increased canopy closure with time. Plant height may have served an important trait in determining the growth of the species under the growing canopy of the stands. Our result is consistent with that of Tripathi et al. [4], who reported that a fast-growing and deciduous *Acacia* species showed the highest growth rate in high-light conditions among species planted under different forest-canopy conditions. However, the result of the present study is in contrast to the results in Choi et al. [31], i.e., above-ground growth of *Z. serrata* seedlings increased with decreases in light intensity. This discrepancy between these studies was probably because of the differences in light intensity and the observed interaction effects of stand × soil on most of the growth parameters measured in *Z. serrata.* This effect of soil characteristics can be seen in the PCA biplot, in which the growth performance of *Z. serrata* are influenced by both soil characteristics and light availability. Specifically, the growth of *Z. serrata* seedlings planted in the pine stand using RY soil (ZRO) is strongly and positively correlated with light availability and soil temperature, which are negatively correlated with all the soil variables. This could explain the decrease in relative height growth of *Z. serrata* seedlings planted in RY soil in the oak stand and a reverse effect when planted in the pine stand. Thus, we can deduce that higher and faster growth is possible for *Z. serrata* seedlings even in nutrient-deficient, sandy-soil conditions, provided that sufficient light is available. This can also explain the observed higher biomass growth of *Z. serrata* seedlings when planted in well-drained, porous GB soil in the pine stand. Our result confirms the findings that *Z. serrata* grows naturally in the lowlands with either full sunlight or partial shade and prefers well-drained alkaline soil [32].

In this study, both height and RCD growth of all species generally increased with time in all forest stands and soil types. This implies that all the species studied were able to adapt and respond positively to fluctuating or contrasting light and soil conditions, but with varying growth performance depending on how they change with time. This is because dependence on light, for example, may have been either gradual or abrupt depending on the species’ life-history traits (e.g., fast-growing vs. slow-growing). Here, the seedlings of the two fast-growing, deciduous species were affected by both stand and soil characteristics, whereas the slow-growing, evergreen species were mostly associated with soil characteristics as shown in their loadings in the PCA biplot. This suggests that the magnitude of the treatment effects may have also been influenced by the contrasting life-history traits.

## 4. Materials and Methods

### 4.1. Study Site

A 3-year intersite experiment was carried out from 2015 to 2017 at the experimental forest at Chungnam National University: one at the oak stand (36°22′13.68″ N, 127°20′33.07″ E) and the other at pine stand (36°22′11.52″ N, 127°20′32.55″ E). Oak stand is dominated by deciduous *Quercus acutissima* Carruth, whereas pine stand is dominated by evergreen *Pinus rigida* Mill. (Table 2). *Q. acutissima* has higher DBH and lower tree height than *P. rigida.* Tree density was higher in pine stand but with a lower basal area than oak stand. Based on our observation and tree-height data, we could say that the ratio of canopy depth/tree height may be higher in the oak stand than the pine stand. Thus, the forest canopy at the pine stand is generally more open (c.a., 30–45%), and therefore receives more light than oak stand throughout the year (Figure S1). In Korea, *Q. acutissima* drops litter both in the spring and fall seasons [33]. Contrarily, the peak time of leaf-litterfall production in *P. rigida* was observed in October–November and in June–July for reproductive-part litterfall [34].

**Figure 6:**
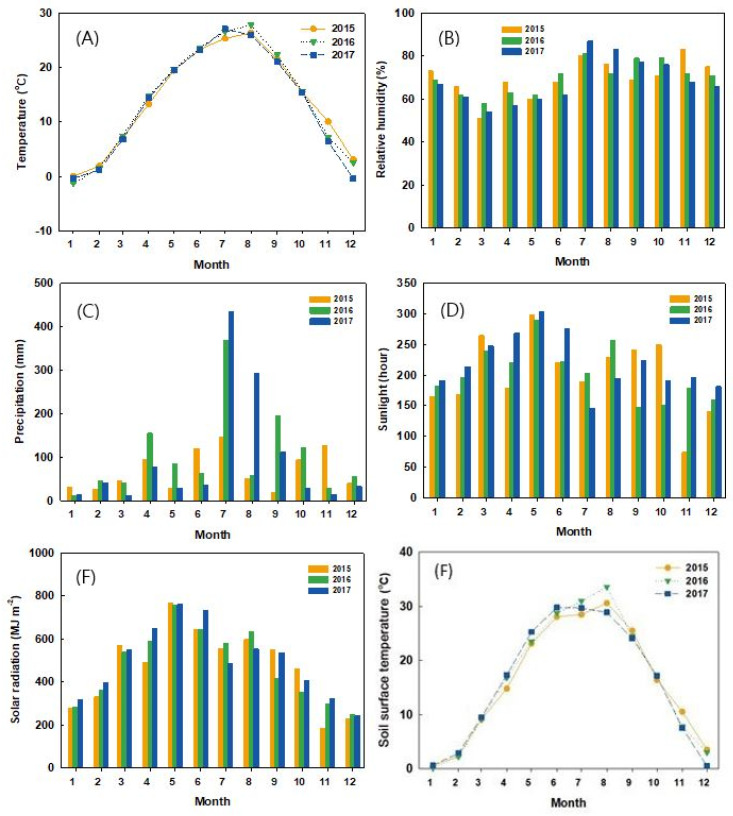
Environmental variables ((**A**) monthly average air temperature, (**B**) monthly average relative humidity, (**C**) monthly precipitation, (**D**) monthly sunlight, (**E**) monthly solar radiation, (**F**) monthly average soil-surface temperature) measured close to the study sites. All variables were measured at the Daejeon Meteorological weather station (36°22′19.2″ N 127°22′19.6″ E) from 2015 to 2017.

The environmental data used in the study were obtained from a weather station close to the experimental sites (1 km in distance, 36°22′19.2″ N 127°22′19.6″E) (Figure 6). Mean annual temperature of the two stands was 13.79 °C, with the highest and lowest monthly temperature of −1.3 °C to 0 °C in January (winter) and 25.4 °C–27.1 °C in July (summer), respectively. The mean monthly precipitation was 88 mm and the highest precipitation rate was also observed during summer, particularly in July (145.6 mm–434.5 mm). Mean annual relative humidity was 69.4%, with the highest values obtained in the summer and fall season (July–November). Mean solar radiation was 481 MJ m^−2^, with the highest radiation (759 MJ m^−2^–769 MJ m^−2^) detected in May in all the experimental periods. Soil-surface temperature, with a mean annual value of 16 °C, was also at its peak during summer (28.13 °C–29.48 °C) and started to decline from fall towards winter. The same pattern was observed in soil temperature at 10 cm depth across experimental periods.

### 4.2. Plant Materials and Experimental Design

One-year-old nursery-grown seedlings of the three forest-tree species with contrasting life-history and/or ecophysiological traits were used in this study (Table 3), two of which are broadleaved deciduous (i.e., *F. rhynchophylla* and *Z. serrata*), while the other is a needle-leaf evergreen tree species (i.e., *P. koraiensis*). All species occur in a range of different habitats from low to high elevations. In terms of light requirement, both *F. rhynchophylla* and *P. koraiensis* are in between full-sun and shade-tolerant, whereas *Z. serrata* is a heliophilous species or well-adapted to full sunlight. In addition to their contrasting characteristics, these species were selected because of their high economic and ecological importance in temperate forests, particularly in East Asia.

Seedlings of the three species were planted in 45-L pots, with a depth of 49 cm and an inner surface diameter of 36 cm. We used 60 pots for each forest stand and each pot was filled with both A- and B-soil horizons from oak and pine stands to maintain the original soil layer. To conduct the intersite experiment, potted seedlings were grown in two contrasting forest stands (i.e., oak vs. pine) using two different soil types, i.e., gray-colored sandy loam (GB) from the oak stand and dark-red-colored sandy clay loam (RY) from the pine stand. Thus, the treatments in the present study were the two types of forest stands and the two different soil types.

Five plots were established in each forest stand, each consisting of three species, two soil types, and two pots per plot. Thirty pots, or half of the total number of pots, were filled with soil from the oak stand and the remaining pots were filled with soil from the pine stand. No additional soil or amendment from the other sources was added in the pots. Some potted seedlings were placed in the prepared holes to have the same level with the forest floor in all stands. The average distance between pots was 2 m × 3 m and was randomly distributed in each plot, while the distance between rows was 2 m. The two forest stands were approximately 100 m away from each other. During the acclimatization period, the seedlings were watered using 10 L/pot/day for several days until treatment imposition. Manual weeding and spraying of pesticide were also performed every year in each stand.

### 4.3. Soil Analysis

In 2015 at 8:00 to 10:00 a.m., soil samples were collected from three randomly selected points per plot in the study site. Samples were categorized into soil horizon A (i.e., 0–25 cm depth) and horizon B (i.e., 25–50 cm depth) by soil color. After drying at 65 °C for 48 h in the oven, the physical and chemical properties of the composited soil samples (*n* = 5) by horizon per plot were analyzed. The soil pH was determined using a pH-meter method, organic matter (OM) and total nitrogen (TN) by the dry-oxidation (utilizing C-N analyzer equipment) method, and available phosphorus (P) with the Lancaster method. The other soil properties such as cation-exchange capacity (CEC), electrical conductivity, and exchangeable K^+^, Ca^2+^, and Mg^2+^ were analyzed using the 1N-ammonium acetate-replacement-leaching method, EC-meter (1:5 soil-water) method, and 1N-ammonium acetate leaching with the atomic absorption spectrophotometry method, respectively.

### 4.4. Light Measurement

Light measurement in lux (converted to a percentage) was conducted in each forest stand from March (spring season) to November (fall season). The measurement was taken once a week, 1–2 h before noon in the three pre-identified points in each forest stand using a handheld light meter (TENMARS TM-202 Lux/fc, Taipei, Taiwan). An average value of 3–5 readings was recorded and taken only during sunny days from each point. Relative light availability (%) was calculated by values at each stand divided by values at open space between two stands. During the measurement, we made sure that the light meter sensor was facing directly upwards in order to accurately detect the amount of light reaching the sensor from the sky hemisphere. The distance between the oak and pine stands was within a distance of 200 m, so the time between light measurements did not exceed ten minutes.

### 4.5. Growth Measurement and Analysis

Tree-seedling height and root-collar-diameter (RCD) growth were measured yearly after planting, while the biomass growth was measured at the harvesting time in 2017. Measurements of RCD and height of seedlings were carried out using a digital caliper and foldable ruler, respectively. Seedling height was measured from the RCD to the highest terminal bud. At the end of the growing season in 2017, the seedlings were harvested for biomass-growth measurement. Relative growth rates were also analyzed for each species. Harvested seedlings were separated into leaf, stem, and root components. The above-ground and below-ground biomasses were determined using the oven-drying method (i.e., 65 °C for 48 h).

### 4.6. Statistical Analysis

All the statistical analyses were conducted in the R Statistical Package Software at a 95% confidence level. Two-way repeated analysis of variance (ANOVA) with post hoc test was carried out for each species to evaluate the treatment and interaction effects on RCD and height growth between oak and pine stands and between soil types with three years of repeated measures. Two-way ANOVA with post hoc test was carried out for each species to evaluate the treatment and interaction effects on biomass growth between oak and pine stands and between soil types. Principal component analysis (PCA) was also run to analyze the relationship among growth, soil, and stand characteristics for all species using the *prcomp* function. Only principal components (PCs) with ≥1 eigenvalues were considered in the biplot using the *factoextra* and *devtools* R packages.

## 5. Conclusions

It is difficult to determine which of the complexities of different canopy characteristics of the forest stands and light environments are more important for seedling growth in this study; however, we revealed that the seedling growth of fast-growing and deciduous *F. rhynchophylla* and *Z. serrata* were higher in the pine stand or in sites with higher light availability than the oak stand or in sites with lower light availability. The seedling growth of slow-growing and evergreen *P. koraiensis* was not impacted by both light and soil conditions. Lastly, the significant growth effects of soil attributes were observed only in the seedlings of *Z. serrata* in both forest stands. The present study recommends further studies to consider the effects of the other traits influencing the plant responses and/or environmental factors affecting the actual environment in the forest floor.

## Figures and Tables

**Table 1:** Soil properties from oak and pine stands. Parentheses are standard errors of the mean (*n* = 5).

Soil Horizon	A Horizon				B Horizon			
Forest Type	Oak Stand		Pine Stand		Oak Stand		Pine Stand	
	(GB)		(RY)		(GB)		(RY)	
Soil texture								
Sand (%)	74.3	(2.2)	62.3	(1.9)	77.9	(3.2)	58.1	(1.0)
Silt (%)	19.4	(2.4)	26.7	(1.2)	16.2	(0.8)	27.2	(1.3)
Clay (%)	6.3	(0.7)	10.9	(0.8)	5.9	(2.5)	14.7	(0.4)
Organic matter (%)	2.3	(0.3)	4.8	(0.3)	0.5	(0.1)	2.0	(0.3)
pH	5.43	(0.12)	4.87	(0.09)	5.57	(0.09)	4.93	(0.07)
Total nitrogen (%)	0.13	(0.01)	0.16	(0.00)	0.05	(0.01)	0.09	(0.01)
Available phosphorus (mg kg^−1^)	17.67	(3.53)	14.33	(2.85)	7.00	(1.00)	5.67	(0.88)
Exchangeable K^+^ (cmol_c_ kg^−1^)	0.34	(0.02)	0.11	(0.01)	0.21	(0.03)	0.10	(0.02)
Exchangeable Ca^2+^ (cmol_c_ kg^−1^)	1.56	(0.34)	1.10	(0.11)	1.23	(0.17)	0.82	(0.06)
Exchangeable Mg^2+^ (cmol_c_ kg^−1^)	0.67	(0.02)	0.22	(0.03)	0.41	(0.06)	0.14	(0.04)
Exchangeable Na^+^ (cmol_c_ kg^−1^)	0.10	(0.01)	0.08	(0.01)	0.09	(0.01)	0.11	(0.01)
CEC (cmol_c_ kg^−1^)	6.82	(0.46)	10.01	(0.44)	4.03	(1.18)	8.91	(0.32)

**Table 2:** The forest structure of oak and pine stands in the experimental forest at Chungnam National University, South Korea. Parentheses are standard errors of the mean (*n* = 5).

Stands	Dominant Species	Average Height (m)	DBH (cm)	Density (Trees ha^−1^)	Basal Area (m^2^ ha^−1^)
Oak	*Quercus acutissima*	13.7 (2.0)	39.8 (11.2)	500 (33)	78 (15)
Pine	*Pinus rigida*	18.1 (1.8)	26.7 (1.8)	800 (58)	47 (8)

**Table 3:** Contrasting life-history/ecological traits of *Fraxinus rhynchophylla*, *Pinus koraiensis*, and *Zelkova serrata*.

Plant Traits	Species		
	*Fraxinus rhynchophylla*	*Pinus koraiensis*	*Zelkova serrata*
Functional type	Broadleaf deciduous	Needle-leaf evergreen	Broadleaf deciduous
Growth rate	Semi-fast-growing	Slow-growing	Fast-growing
Native range	East Asia (temperate)	East Asia (temperate)	East Asia (temperate)
Mature size	DBH: 50–60 cm; height: 25–30 m [23]	DBH: >50 cm; height: >22–40 m [35]	DBH: 50–60 cm; height: 20–25 m
Seed dispersal	By wind	By animals (e.g., birds, squirrels, and rodents) [36]	Seed-bearing shoot [37]
Seed/cone size/mass	Very small winged seed (c.a. 10 g/300 pcs)	Large (500–600 mg) [38]	1 g/90 shoot-seeds
Seedling architecture	Epigeal	Epigeal	Epigeal
Habitat requirements	Moist, fertile soils; hillsides and river valleys [39]	Deep fertile soil; wide range of rainfall levels [40]	Tolerates most soil types, with pH of about 7.5, moist, and well-drained soils
Light requirement	In between full-sun and shade-tolerant [23]	In between full-sun and shade-tolerant [41]	Full sunlight

## Data Availability

Data is contained within the article or Supplementary Materials.

## Supplementary Materials

The following supporting information can be downloaded at: https://www.mdpi.com/article/10.3390/plants11070841/s1, Figure S1: crown structure of (A) pine stand and (B) oak stand; Figure S2: Relative height growth rate of *Fraxinus rhynchophylla*, *Pinus koraiensis*, and *Zelkova serrata* in different soil and forest stands from 2015 to 2017; Figure S3: Relative root collar diameter growth of *Fraxinus rhynchophylla*, *Pinus koraiensis*, and *Zelkova serrata* in different soil and forest stands from 2016 to 2017; Table S1: *p* values estimated by two-way analysis of variance (ANOVA) for soil physical and chemical properties across soil horizons and stands; Table S2: *p* values estimated by two-way repeated-measures analysis of variance (ANOVA) for height growth and RCD growth of three species at harvesting year across soil horizons and stands; Table S3: *p* values estimated by two-way analysis of variance (ANOVA) for biomass growth of three species across soil properties and stands.
