# Supplementary material for: Growth of Deciduous and Evergreen Species in Two Contrasting Temperate Forest Stands in Korea: An Intersite Experiment
Source: Plants (Basel). 2022 Mar 22;11(7):841. doi: 10.3390/plants11070841 (PMC9002389; doi:10.3390/plants11070841)
Supplement: Supplementary file 1 [file plants-11-00841-s001.zip › plants-1618485-supplementary.pdf]

Supplementary Materials

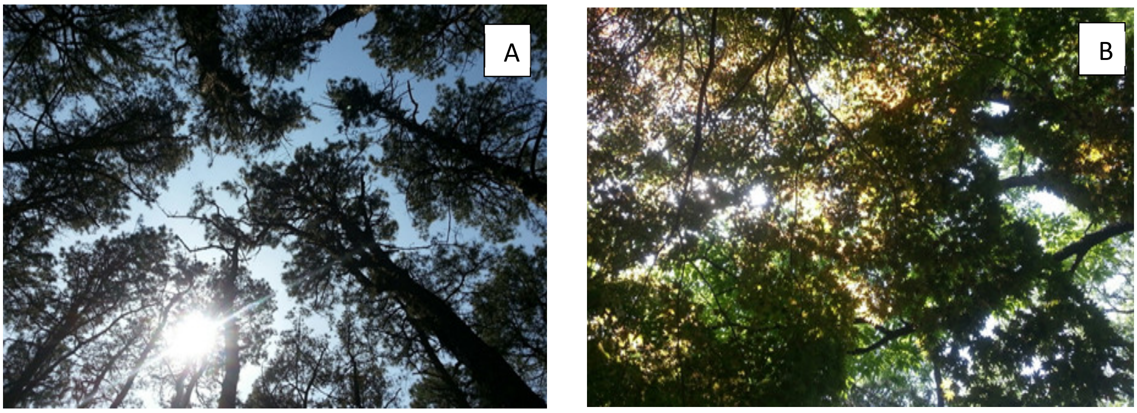

Figure S1. Crown structure of (A) pine stand and (B) oak stand.

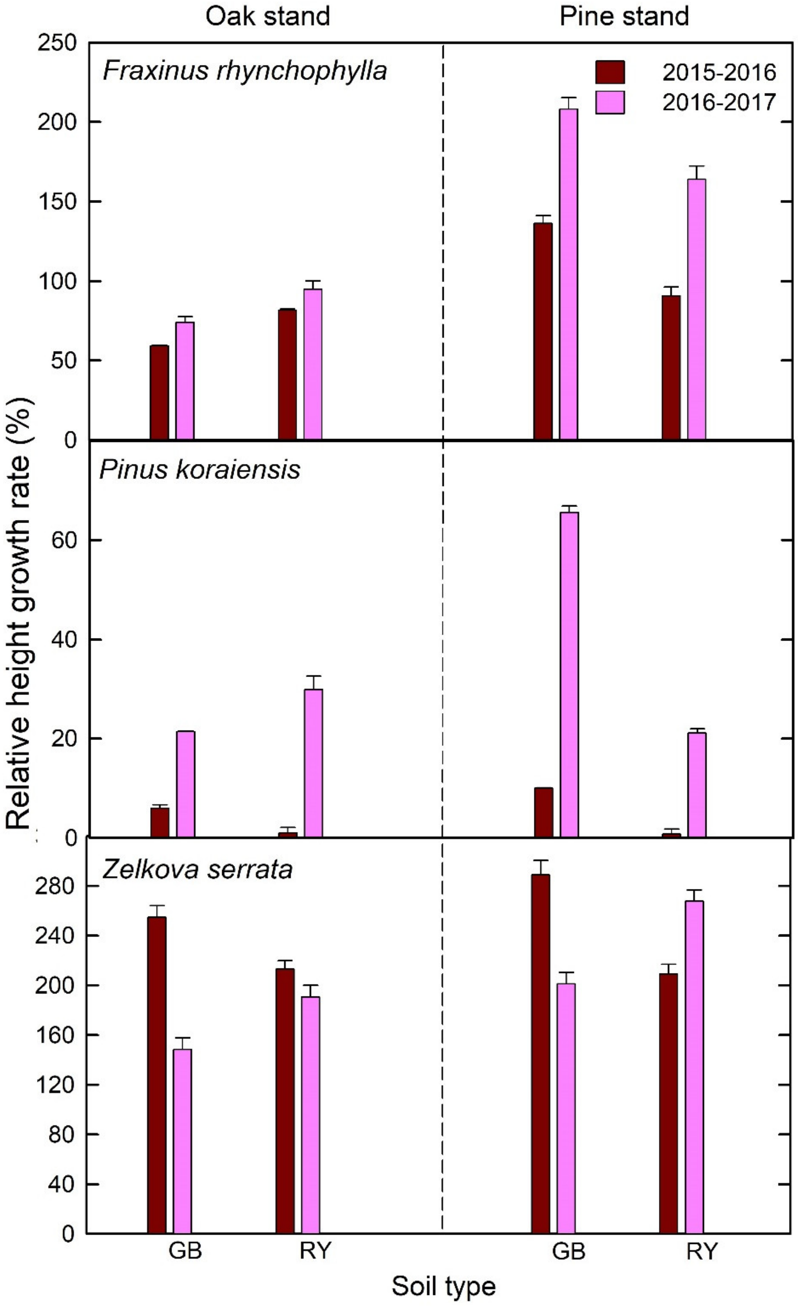

Figure S2. Relative height growth rate of *Fraxinus rhynchophylla*, *Pinus koraiensis*, and *Zelkova serrata* in different soil and forest stands from 2015 to 2017.

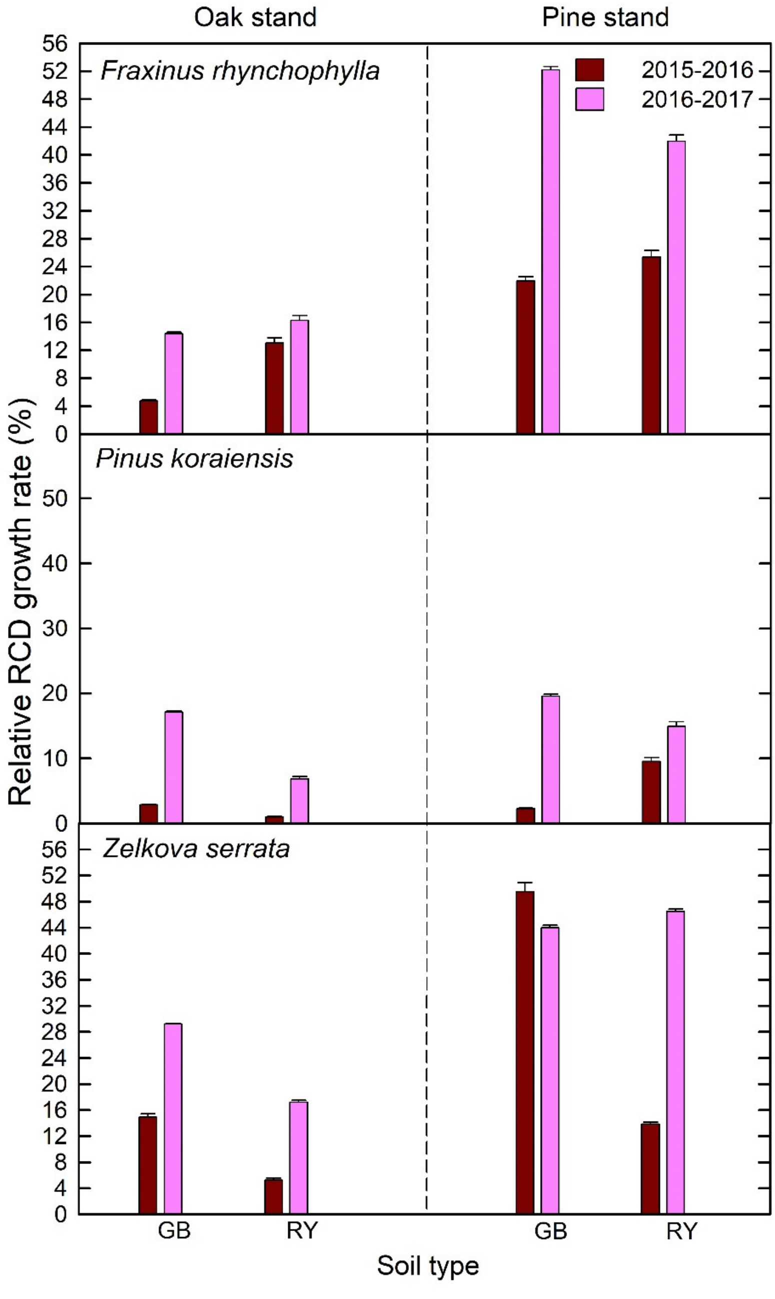

Figure S3. Relative root collar diameter growth of *Fraxinus rhynchophylla*, *Pinus koraiensis*, and *Zelkova serrata* in different soil and forest stands from 2016 to 2017.

Table S1. P values estimated by two-way analysis of variance (ANOVA) for soil physical and chemical properties across soil horizons and stands.

Source of variables	Sand		Silt		Clay		OM		pH		Total N	
	F value	Pr > F	F value	Pr > F	F value	Pr > F	F value	Pr > F	F value	Pr > F	F value	Pr > F
Stand	52.45	**<0.001**	34.90	**<0.001**	23.58	**0.001**	51.62	**<0.001**	41.81	**<0.001**	18.12	**0.003**
Soil horizon	0.02	0.895	0.73	0.417	1.44	0.265	68.53	**<0.001**	1.16	0.313	82.29	**<0.001**
Stand × Soil horizon	3.16	0.114	1.39	0.273	2.29	0.169	4.17	0.075	0.13	0.729	0.00	0.985

Source of variables	Available P		Exchangeable K^+		Exchangeable Ca^{2+}		Exchangeable Mg^{2+}		Exchangeable Na^+		CEC	
	F value	Pr > F	F value	Pr > F	F value	Pr > F	F value	Pr > F	F value	Pr > F	F value	Pr > F
Stand	0.98	0.352	62.30	**<0.001**	4.78	0.060	74.01	**<0.001**	0.13	0.733	34.23	**<0.001**
Soil horizon	16.74	**0.004**	10.56	**0.012**	2.34	0.165	15.41	**0.004**	1.13	0.320	7.95	**0.024**
Stand × Soil horizon	0.18	0.683	8.65	**0.019**	0.02	0.889	4.42	0.069	3.13	0.115	1.50	0.256

Table S2. P values estimated by two-way repeated measures analysis of variance (ANOVA) for height growth and RCD growth of three species at harvesting year across soil horizons and stands.

		Height growth		RCD growth	
Species	Source	F Value	Pr>F	F Value	Pr>F
Fraxinus rhynchophylla	Time	104.48	**<0.001**	177.95	**<0.001**
	Stand	20.65	**<0.001**	70.99	**<0.001**
	Time × stand	9.29	**0.001**	42.67	**<0.001**
	Soil	0.55	0.466	0.01	0.935
	Time × soil	0.16	0.851	0.59	0.555
	Stand × soil	2.69	0.112	2.55	0.122
	Time × Stand × soil	1.59	0.213	1.30	0.280
Pinus koraiensis	Time	12.45	**<0.001**	27.80	**<0.001**
	Stand	0.11	0.747	5.93	0.035
	Time × stand	0.98	0.381	1.49	0.250
	Soil	0.01	0.936	0.26	0.621
	Time × soil	1.44	0.263	0.87	0.433
	Stand × soil	2.28	0.162	3.18	0.105
	Time × Stand × soil	1.60	0.230	0.58	0.572
Zelkova serrata	Time	132.56	**<0.001**	60.67	**<0.001**
	Stand	10.23	**0.008**	25.42	**<0.004**
	Time × stand	1.53	0.239	9.72	**<0.001**
	Soil	6.35	**0.029**	11.17	**0.007**
	Time × soil	0.53	0.591	4.34	**0.026**
	Stand × soil	3.21	0.101	5.60	**0.037**
	Time × Stand × soil	0.22	0.807	0.67	0.521

Table S3. P values estimated by two-way analysis of variance (ANOVA) for biomass growth of three species across soil properties and stands.

Species	Source of variables	Leaf	Stem	Fine root	Coarse root	Total biomass	Root to shoot ratio
Fraxinus rhynchophylla	Stand	**<0.001**	**<0.001**	**<0.001**	**<0.001**	**<0.001**	**<0.001**
	Soil type	0.741	0.618	0.197	0.626	0.420	0.209
	Stand × Soil type	0.209	0.249	0.133	0.228	0.175	0.935
Pinus koraiensis	Stand	0.086	0.058	0.319	**0.042**	0.295	**0.001**
	Soil type	0.423	0.451	0.666	0.341	0.625	0.086
	Stand × Soil type	0.364	0.269	0.586	0.228	0.237	0.116
Zelkova serrata	Stand	0.125	**0.009**	**0.005**	**<0.001**	**0.005**	**0.018**
	Soil type	0.093	**0.025**	**0.029**	0.302	**0.032**	**0.042**
	Stand × Soil type	0.155	**0.041**	**0.028**	0.124	**0.041**	0.494
